# Presence of intratumoral platelets is associated with tumor vessel structure and metastasis

**DOI:** 10.1186/1471-2407-14-167

**Published:** 2014-03-10

**Authors:** Rong Li, Meiping Ren, Ni Chen, Mao Luo, Xin Deng, Jiyi Xia, Guang Yu, Jinbo Liu, Bing He, Xu Zhang, Zhuo Zhang, Xiao Zhang, Bing Ran, Jianbo Wu

**Affiliations:** 1 Drug Discovery Research Center, Luzhou Medical College, Luzhou, Sichuan, People's Republic of China; 2 Department of Physiology, Luzhou Medical College, Luzhou, Sichuan, People's Republic of China; 3 Dalton Cardiovascular Research Center, University of Missouri, Research Park Dr., Columbia 652121, MO, USA

**Keywords:** Platelets, Tumorigenesis, Metastasis, Hypoxia, Angiogenesis

## Abstract

**Background:**

Platelets play a fundamental role in maintaining hemostasis and have been shown to participate in hematogenous dissemination of tumor cells. Abundant platelets were detected in the tumor microenvironment outside of the blood vessel, thus, platelet -tumor cell interaction outside of the bloodstream may play a role in regulating primary tumor growth and metastasis initiation. However, it is unclear that platelet depletion affects tumor vessel structure and dynamics.

**Methods:**

Using thrombocytopenia induction in two different tumor-bearing mouse models, tumor tissues were performed by Westernblotting and immunohistochemical staining. Vascular permeability was evaluated by determination of intratumoral Evans blue and Miles vascular permeability assay. Furthermore, microdialysis was used to examining the intratumoral extracellular angiogenic growth factors (VEGF, TGF-β) by ELISA.

**Results:**

Platelet depletion showed no change in tumor growth and reduced lung metastasis. Platelet depletion led to reduced tumor hypoxia and Met receptor activation and was associated with a decreased release of MMP-2, 9, PAI-1, VEGF, and TGF-β. Tumor vessels in platelet-depleted mice showed impaired vessel density and maturation.

**Conclusions:**

Our findings demonstrate that platelets within the primary tumor microenvironment play a critical role in the induction of vascular permeability and initiation of tumor metastasis.

## Background

In addition to hemostasis, circulating platelets play a vital role in tumor progression and metastasis [1-3]. The proposed molecular mechanisms mainly involve the hematogenous dissemination *of* tumor cells. Platelet interaction with tumor cells is known to contribute to metastasis by shielding tumor cells from NK cell destruction, aiding endothelial attachment, releasing angiogenic and growth factors such as vascular endothelial growth factor (VEGF) and tumor growth factor-β (TGF-β), and assisting tumor cell invasion. Experimental evidence suggests that the depletion of platelets results in anti-tumor dissemination in thrombocytopenic mice [4-7]. The leaky tumor vasculature allows platelets to come in contact with the tumor and deposit multiple angiogenic factors near tumor cells, which in turn contribute to tumor progression. A recent study demonstrated that abundant platelets were detected in the primary tumor microenvironment away from the vasculature [7], and thus, it is likely that the pro-metastatic role of platelets is not limited to circulating dissemination.

The tumor microenvironment is critical in facilitating tumor growth and metastasis, and hypoxia of the microenvironment is believed to directly affect the ability of tumor cells to metastasize [8,9]. The role of platelets in tumor angiogenesis and the modulation of vessel permeability are well established, whereas their effect on the tumor microenvironment is still undefined. It has been proposed that platelets may play a direct role in the mobilization of primary tumor cells to vessels for metastasis. However, there has been no direct evidence for how platelets cause increased local invasion. Previous studies demonstrated that the depletion or reduction of circulating platelets resulted in reduced experimental metastasis of various tumors [3,7,10,11], and the requirement of functional platelets for circulating tumor dissemination has been confirmed in many experimental settings. The current study was designed to test the hypothesis that platelets influence metastasis by mediating tumor vessel structure and dynamics.

## Methods

### Animals

All animal procedures described in this study were performed using 6- to 8-wk-old C57BL/6 J mice or BALB/c mice (purchased from The Jackson Laboratory). Animal use was approved by the Animal Experimentation Committee of Luzhou Medical College.

### Cell culture

Murine B16/F10 melanoma cells or 4 T1 mouse mammary epithelial cancer cells were obtained from American Type Culture Collection (Manassas, VA, USA), and grown in DMEM media supplemented with 10% fetal calf serum (FCS), 100 U/ml penicillin and 100 U/ml streptomycin.

### Tumor cell implantation

Mice were anesthetized with ketamine/xylazine, and 1X10^6^ B16/F10 melanoma cells or 4 T1 mouse breast cancer cells (8 mice /each group) were implanted subcutaneously in the back. Tumor volumes were measured every 3 days using Vernier calipers, and volumes were calculated using a standard formula (length x width^2^ x 0.52). Mice were sacrificed when tumor growth reached 25 days post-cancer cell implantation.

### Induction of thrombocytopenia

When the average B16/F10 tumor size reached ~ 500 mm^3^, or 4 T1 tumor size reached ~ 250 mm^3^, thrombocytopenia was induced by intraperitoneal (i.p.) injections every 3 days of 2.5 μg/g mouse platelet-depleting antibody (polyclonal anti-mouse GPIbα rat IgG; emfret Analytics). Control mice were injected with a nonimmune rat polyclonal IgG (emfret Analytics). Thrombocytopenia was evaluated by blood count. The i.p. injection of the depleting antibody resulted in ≥95% reduction in circulating platelets at 12 h post-injection in all mice.

### Quantification of metastasis

The metastatic area of lung was quantified as described previously [12]. Briefly, HE staining of paraffin-embedded lung sections was performed, and light photomicrographs were taken from the bilateral lobe of the lung and reconstructed using the Adobe Photoshop CS4 function. Metastases were identified via hispathological analysis, and the metastatic area was quantified as a percentage of the total reconstructed lung area using NIH ImageJ software. High-magnification images of the metastatic area were obtained by magnifying the original images by 40×.

### Immunoblotting

Tumor tissues were homogenized in RIPA buffer (Sigma). Equal amounts of protein were subjected to SDS-PAGE and transferred to polyvinylidene difluoride membranes by electroblotting. After blocking, the membranes were incubated with phosphospecific and nonphosphospecific antibodies directed against Met, HIF-1α, Angiopoietin 1, Angiopoietin 2, VE-cadherin, PAI-1, MMP-9, and β-actin. Relative band density for Western blotting was determined using ImageJ gel analysis software.

### Immunofluorescence and quantification

To quantify pericyte coverage (α-SMA, red channel), we drew a region of interest (ROI) close to each blood vessel (PECAM-1, green channel) and calculated the mean fluorescence intensity of the red and green channels using the Zeiss Confocal Software Histogram Quantification Tool. Values were expressed as a percentage of red to green. Quantification was performed by analyzing at least 3 sections and 3 fields per tumor.

### Quantification of VEGF and TGF-β levels

Microdialysates were analyzed for VEGF and TGF-β protein content using commercial quantitative immunoassay kits (R&D Systems). The analyzed proteins were normalized to the total protein content and expressed as pg/mg protein.

### Tumor hypoxia analysis

Tumor hypoxia was quantified as described previously [13]. Tumor tissues were collected 2 hours after injection of 60 mg/kg pimonidazole hydrochochloride (HP2100 Hypoxyprobe Kit-Plus; Natural Pharmacia International Inc.) into mice. The formation of pimonidazole adducts was detected by immunostaining with Hypoxyprobe-1-Mab 1 antibody according to the manufacturer’s instructions. Images were captured and analyzed using an Olympus (DP70) microscope and then evaluated using the the Adobe Photoshop CS4 function. Quantification was performed by analyzing at least 3 sections and 3 fields per tumor.

### Determination of intratumoral Evans blue

Mice were injected i.v. with 100 μL 5% Evans blue. Three hours after injection, the tumors were isolated from the surrounding tissue, weighed, and placed in 0.5 ml formamide. Three days later, the supernatants were measured by reading the absorbance at 620 nm. Data were presented as micrograms of Evans blue dye per gram of tissue.

### Miles vascular permeability assay

The miles assay was performed as previously described [14]. Briefly, mice were administrated Evans blue dye. VEGF (300 ng in 15 μl) or saline was injected subcutaneously into the dorsal surface of the right and left ears, respectively. After 30 minutes, mice were euthanized and their ears removed, oven-dried at 55°C, and weighed. The Evans blue dye was then extracted from the ears using 500 μl of formamide for 24 hours at 55°C, and the absorbance of extracted dye was measured at 630 nm.

### Tumor perfusion assay

Tumor perfusion assay was performed as previously described in detail [15]. Briefly, Mice were injected with 0.2 ml of 25 mg/ml FITC-dextran (molecular weight 2,000,000; Sigma-Aldrich, St. Louis, MO, USA) by tail vein 20 min before being killed. Whole blood samples were collected and stored at 4°C in the dark. Blood samples were centrifuged at 15000 rpm for 10 min at 4°C and supernatants collected for fluorescence assay. Tumors were harvested, weighed, and treated with dispase (1:10 dilution, 1 ml per 0.5 g tumor tissue) at 37°C in a shaker for 4 h in the dark. Tumor tissues were then homogenized and centrifuged at 16000 rpm for 15 min. Supernatants were collected and stored in the dark at 4°C. Supernatant fluorescence was measured in a reader (Molecular Device, USA). The ratio of tumor fluorescence/plasma fluorescence reflects the extent of tumor blood vessel perfusion.

### Determination of intratumoral hemoglobin content

Tumors were excised from the backs of the sacrificed animals, weighed, homogenized in Drabkin's reagent (Sigma), and centrifuged (2000 × *g*; 10 min). The hemoglobin content in the supernatants was measured by reading the absorbance at 540 nm.

### Microdialysis for protein sampling *in vivo*

Microdialysis was performed as previously described in detail [16]. Briefly, mice were anesthetized, and microdialysis probes (CMA/20, 0.5-mm diameter, PES membrane length 4 mm, 100-kDa cutoff, CMA/Microdialysis) were inserted into tumor tissue sutured to the skin, connected to a CMA/102 microdialysis pump (CMA/Microdialysis) and perfused at 0.6 μL/min with saline (154 mmol/L NaCl) containing 40 mg/mL dextran (Pharmalink). The outgoing microdialysates were collected on ice and stored at −80°C for subsequent ELISA analysis.

### Statistical analysis

Data are presented as the mean ± SEM and were analyzed by ANOVA and by unpaired two-tailed Student's *t* test. *P* values of <0.05 were regarded as statistically significant.

## Results

### Platelet depletion showed no change in tumor growth and reduced lung metastasis

**Figure 1:**
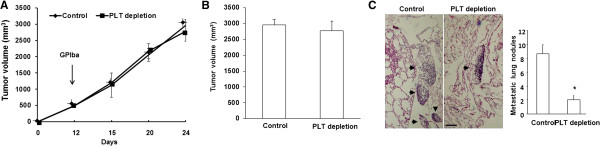
**Platelet depletion showed no change in tumor growth but reduced lung metastasis. (A)** Orthotopic implantation of B16/F10 tumor cells into C57B6/L mice followed by injections every 3 days of GPI or control antibody after the tumors reached ~500 mm^3^. **(B)** Total tumor volumes at the experimental endpoint (24 days). **(C)** Representative images of H&E-stained lung sections. Scale bar, 5 μm. Arrows point to metastatic areas. Number of metastatic lung nodules in the lungs of these mice. Error bars display mean ± SEM; asterisks denote significance (*p < 0.05). **(D)** Western blot analysis of p-Met, total Met, and β-actin expression in tumors from control and PLT-depleted mice. Quantification of western blot analysis for total Met (normalized to β-actin) and p-Met (normalized to β-actin), Error bars display mean ± SEM; asterisks denote significance (*p < 0.05) (*n* = 6 for each group).

Previous studies demonstrated that circulating platelets play a shielding role in cancer cell dissemination and hemorrhagic metastasis [1-3]. To evaluate the role of platelets in primary tumor progression and metastasis, we performed thrombocytopenia induction in a tumor-bearing mice model. B16/F10 melanoma cancer cells were implanted into the backs of C57BL/6 J mice. Primary tumor growth was monitored, and when tumors reached ~500 mm^3^ in size, anti-GPIbα or rat IgG was injected into platelet-depleted or control mice, respectively, every 3 days until 24 days post-injection (Figure 1A). Until the experimental endpoint, platelet-depleted mice showed no change in tumor growth (2777 ± 300 mm^3^*Vs.* 2956 ± 180 mm^3^, p > 0.05) (Figure 1A, B) compared to control mice, while B16/F10 tumor-bearing platelet-depleted mice exhibited a significant reduction in lung metastasis compared to control mice (Figure 1C).

To further investigate whether PLT depletion leads to reduced metastasis in other tumor types, we subcutaneously implanted 4 T1 mouse mammary epithelial cancer cells into BALB/c mice. The mice were then treated with anti-GPIba or rat IgG, respectively, when tumors reached 250 mm^3^ in size. Similarly, platelet-depleted mice showed no change in tumor growth compared to control mice (Additional file 1: Figure S1A). PLT-depleted tumors demonstrated large dark cores, associated with increased hemorrhagic areas, while 4 T1 tumor-bearing platelet-depleted mice exhibited a significant reduction in lung metastasis compared to control mice (Additional file 1: Figure S1B). These results further support that platelets play a role in tumor metastasis in different types of tumors.

### Platelet depletion reduces blood vessel density, vessel maturation, and perfusion

**Figure 2:**
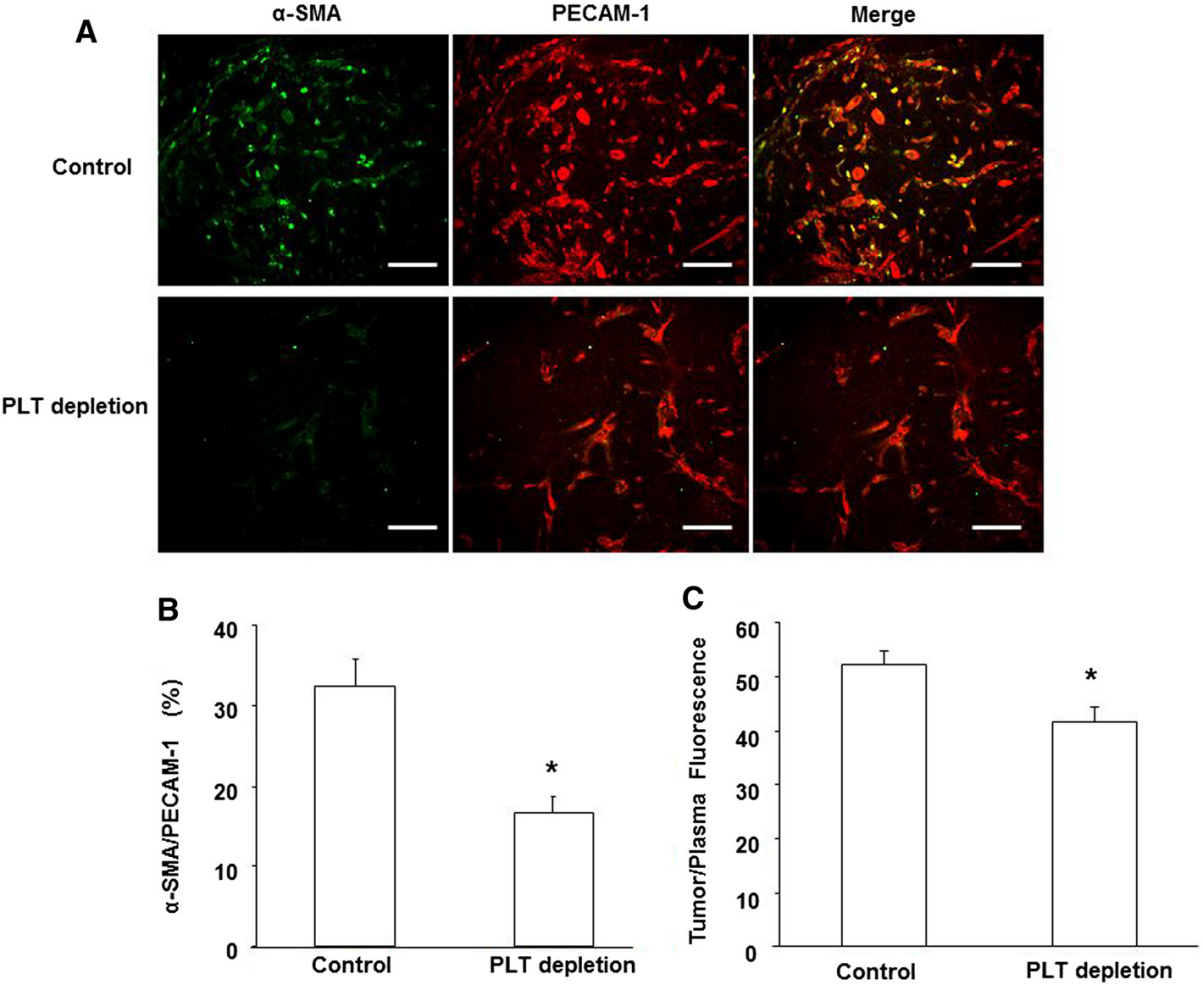
**Platelet depletion reduces tumor blood vessel density, pericyte coverage and perfusion. (A)** Representative images of immunostaining of tumor sections for PECAM-1 (green) and α-SMA (red). Scale bar, 50 μm. Quantitative assessment of PECAM-1-positive blood vessels **(B)** and α-SMA /PECAM-1-positive cells **(C)** in B16/F10 tumors from platelet (PLT)-depleted and control mice. The results were represented as the mean percentage of area ± SME (n = 6). **(D)** Tumor blood vessel perfusion assay using FITC-dextran in vivo. Following 2 weeks of treatment with GPI or control antibody, tumor-bearing mice were injected (iv) with 0.2 ml FITC-dextran (25 mg/ml) for 20 min. Tumors and whole blood were collected, processed, and centrifuged. Fluorescence was measured in the supernatants from tumor tissue and whole body plasma. The ratio of tumor to plasma fluorescence reflects the extent of tumor perfusion. The results are expressed as the mean ± SEM. *p < 0.05. (*n* = 6 for each group).

Because proangiogenic growth factors released from platelet granules could affect tumor vessel formation, we examined vessel density and coverage in the tumors by using anti-PECAM-1 and anti-α–SMA double staining. PECAM-1-positive microvascular density was significantly lower in platelet-depleted B16/F10 tumors compared to control tumors (Figure 2A). Coverage of intratumoral microvessels by α–SMA-positive mural cells was also significantly lower in platelet-depleted B16/F10 tumors compared to control tumors (Figure 2A, B). To better characterize the role of platelets in tumor blood vessel function, we further studied the perfusion of the tumor vasculature in platelet-depleted and control mice. We evaluated the mice based on the ratio of fluorescence intensities within the plasma and tumor following injection with FITC-dextran (MW 20,000). We found that platelet depletion significantly reduced blood vessel perfusion of tumors compared with control mice (Figure 2C).

### Platelet depletion induces vessel leakage

**Figure 3:**
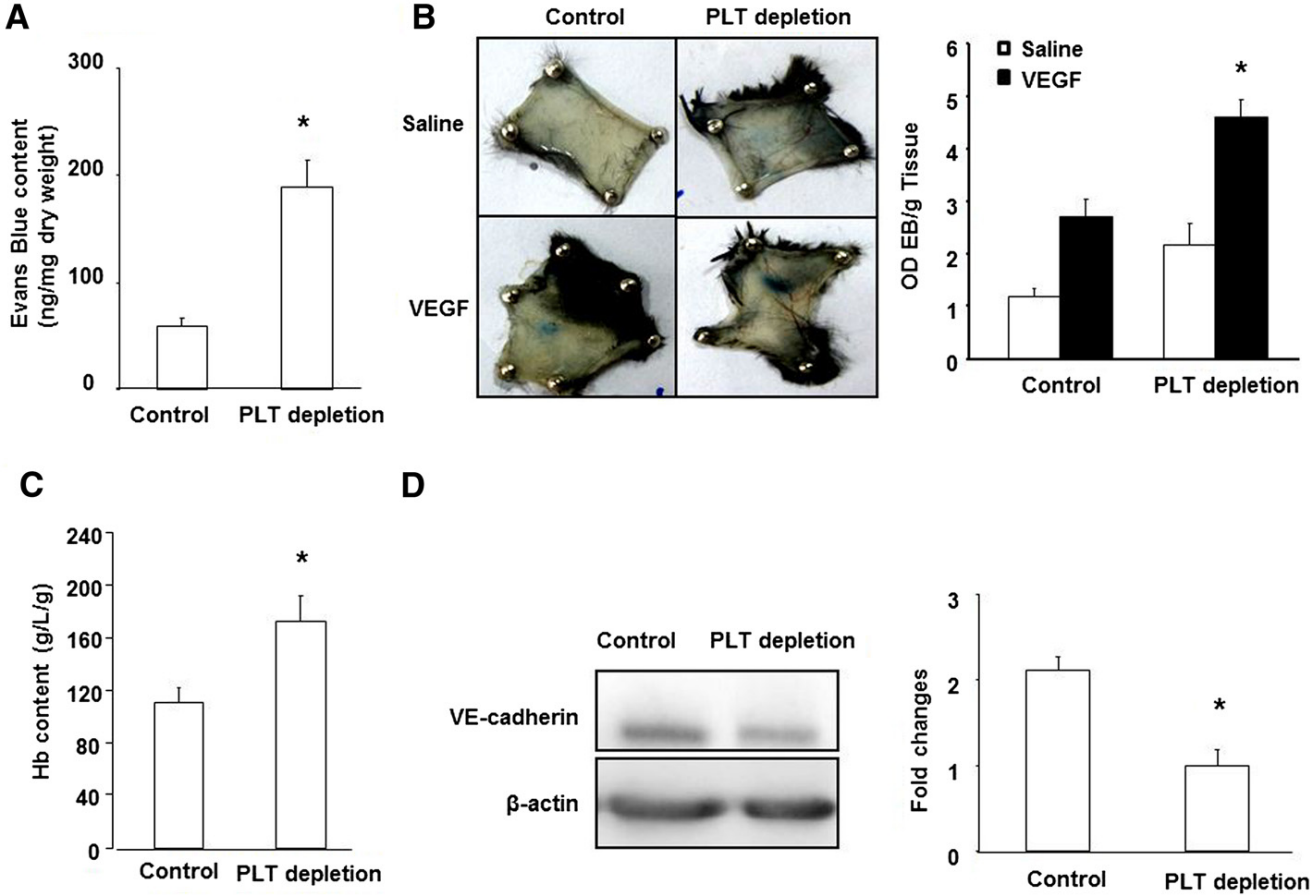
**Platelet depletion increases vessel permeability. (A)** Orthotopic implantation of B16/F10 tumor cells into C57B6/L mice were followed by injections every 3 days of GPI or control antibody after the tumors reached ~500 mm^3^. Evans blue was then injected (100 μL 5% Evans blue, i.v.; 30 min) at the experimental endpoint (24 days). Evans blue was then extracted and quantified spectrophotometrically at 620 nm. *,p < 0.05. **(B)** Photographs of Evan’s blue dye leakage 30 minutes following intradermal injection of either saline or VEGF into the shaved dorsal skin of control and platelet depletion mice. n = 3 for each group. **(C)** Comparison of intratumoral hemoglobin content among control and PLT-depleted mice, *p < 0.05. (*n* = 6 for each group). **(D)** Western blot analysis of VE-cadherin expression in tumors from control and PLT-depleted mice. Quantification of Western blot analysis for VE-cadherin (normalized to β-actin).

Vascular permeability has been correlated to vessel maturation during tumor growth [17,18]. We therefore examined whether the reduced coverage of pericytes to tumor microvessels in platelet-depleted mice affected tumor vascular permeability. Platelet-depleted tumors exhibited a 3.2-fold increase in Evans blue extravasation compared to control tumors (189 ± 25.3 *Vs*. 59.3 ± 7.9 ng/mg tumor dry weight, p < 0.05) (Figure 3A). Furthermore, we used the Miles assay to measure vascular leakage in the skin of control and PLT-depleted mice. As shown in Figure 3B, VEGF-mediated hyperpermeability was significantly increased in the PLT-depleted mice compared with that in the WT mice. Taken together, these results indicated that platelet depletion has an effect in increasing vascular permeability *in vivo*.

We measured the intratumoral hemoglobin content, which reflects the level of erythrocyte extravasation. The hemoglobin content in the tumors of platelet-depleted mice was significantly higher than in control mice (172.11 ± 20.2 g/L/g *Vs*. 110.28 ± 12.4 g/L/g, p < 0.05) (Figure 3C). Collectively, these data strongly suggest that reduced coverage of pericytes in tumor vessels might be another main origin of the increased vascular tumor leakage observed in platelet-depleted mice.To better investigate the molecular mechanisms by which platelet depletion induced vessel leakage in the tumor microenvironment, we evaluated the expression of VE-cadherin protein, which is a critical EC-specific adhesion molecule in regulating vascular permeability. We found that the VE-cadherin protein level was reduced in platelet-depleted tumors compared to controls (Figure 3D), suggesting that platelet depletion-induced vascular leakage is associated with a reduction of VE-cadherin expression.

### Platelet depletion reduced hypoxia, HIF-1α expression, and Met activation

**Figure 4:**
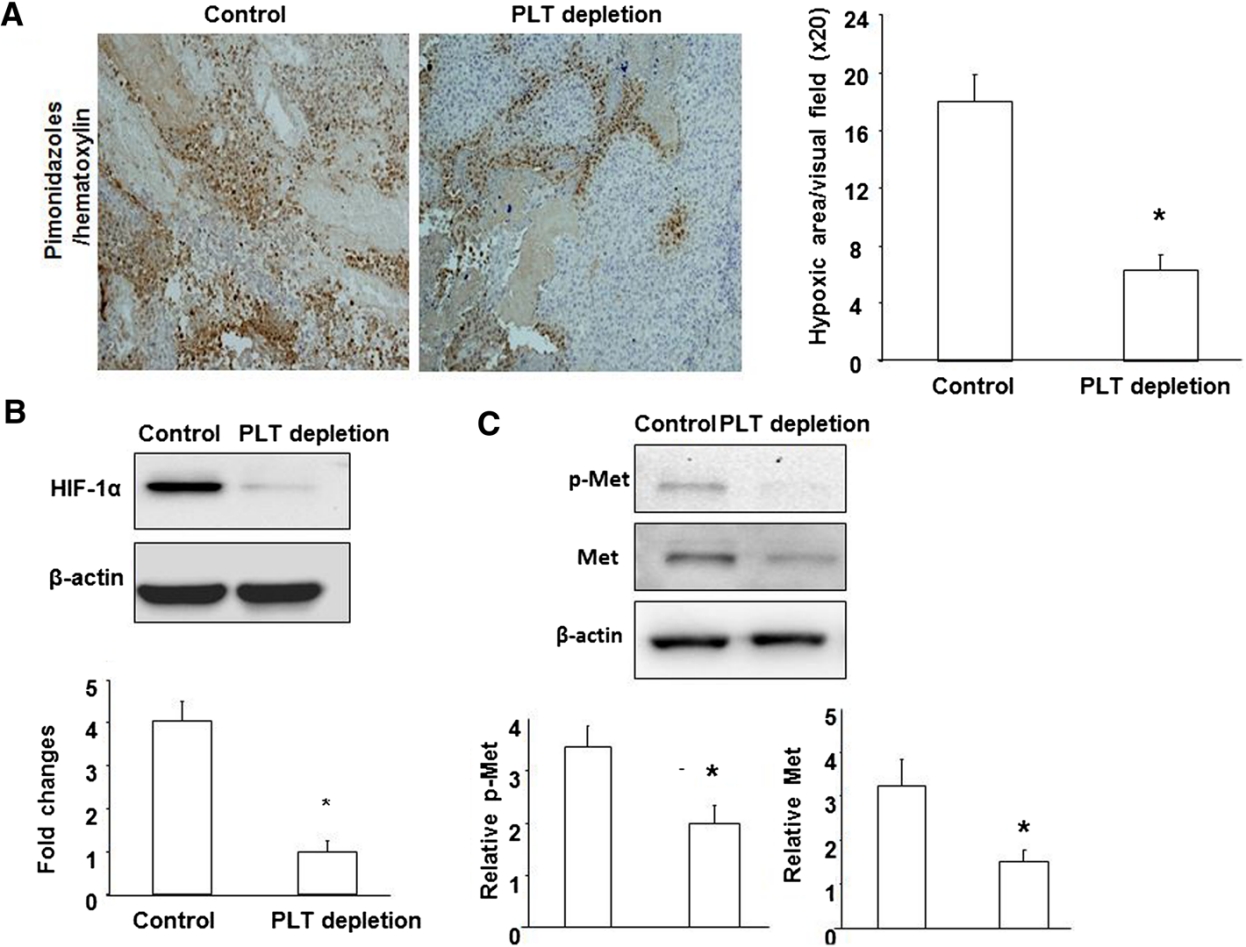
**Platelet depletion reduced hypoxia, HIF-1α expression, and Met activation. (A)** Hypoxia was detected by immunohistochemistry staining of pimonidazole adducts in B16/F10 tumor sections from control and PLT-depleted mice. Nuclei were stained with hematoxylin. Hypoxia was quantified as the percent hypoxic area per visual field. **(B)** Western blot analysis of HIF-1α expression in tumors from control and PLT-depleted mice. Quantification of western blot analysis for HIF-1α (normalized to β-actin). **(C)** Western blot analysis of p-Met, total Met, and β-actin expression in tumors from control and PLT-depleted mice. Quantification of western blot analysis for total Met (normalized to β-actin) and p-Met (normalized to β-actin).

To further gain insight into the molecular mechanisms associated with reduced metastasis resulting from platelet depletion, we first assessed hypoxia levels by examining pimonidazole adduct formation in the tumors of platelet-depleted and control mice and found decreased hypoxic levels in the platelet-depleted tumors (Figure 4A). In addition, expression of the hypoxia-inducible transcription factor HIF-1α was also significantly reduced in the tumors of platelet-depleted mice (Figure 4B), suggesting that platelets are involved in tumor hypoxia.

Based on the known induction effects of hypoxia and cancer invasiveness on the expression and activation of the proinvasive tyrosine kinase receptor Met [12,13], we analyzed total protein and tyrosine phosphorylation levels of Met in both platelet-depleted and control mice. Western blotting analysis revealed that platelet depletion caused a significant decrease of both total Met and phospho-Met in tumors compared to tumors from control mice (Figure 4C).

### Platelets changed intratumoral levels of angiogenic factors

**Figure 5:**
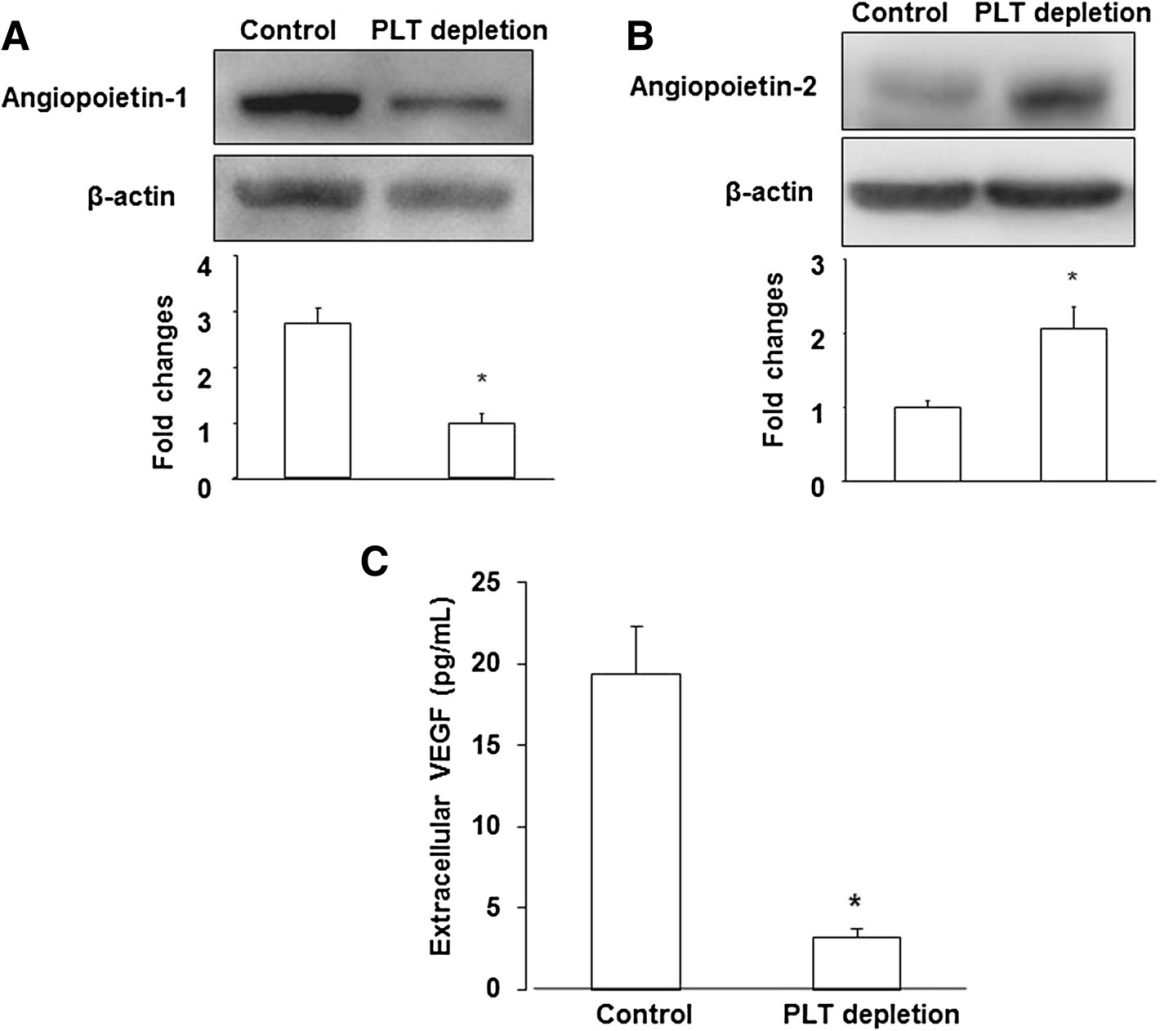
**Platelets changed intratumoral levels of angiogenic factors. (A)**, **(B)** Western blot analysis of Angiopoietin-1, 2 expression in tumors from control and PLT-depleted mice. Quantification of western blot analysis for each protein (normalized to β-actin). **(C)** Microdialysis was used to sample extracellular proteins in vivo. Extracellular VEGF level was measured by ELISA as described in *Materials and Methods*. The results are expressed as the mean ± SEM. * p < 0.05. (*n* = 6 for each group).

Proangiogenic growth factors released from platelet granules can affect tumor cell survival, proliferation, or invasiveness. Because our data indicated decreased hypoxia in platelet-depleted tumors, we considered the possible involvement of growth factors known to regulate tumor growth and metastasis. Previous studies demonstrated that the angiogenic cascade of the Ang-Tie system is critical for controlling vessel assembly and maturation. We performed Western blotting to examine the expression of Ang-1, which induces Tie2 activation leading to vessel stabilization and maturation, and its antagonist Ang-2, which causes vessel destabilization [19,20]. We observed a significant reduction in Ang-1 in platelet-depleted tumors compared to control tumors (Figure 5A). In contrast, the Ang-2 level was significantly increased in platelet-depleted tumors compared to control tumors (Figure 5B). Since the VEGF protein is bioactive as soluble protein in the extracellular space [21], we next examined extracellular VEGF level of tumors. Microdialysis was performed at 24 days before sacrifice, and found a significant decrease of extracellular VEGF in platelet-depleted tumors compared to control tumors (p < 0.05) (Figure 5C). These data suggest that platelets play a role in the secretion of growth factors in the tumor microenvironment.

### Platelets changed levels of TGF-β1, MMP-2,9, and PAI-1

**Figure 6:**
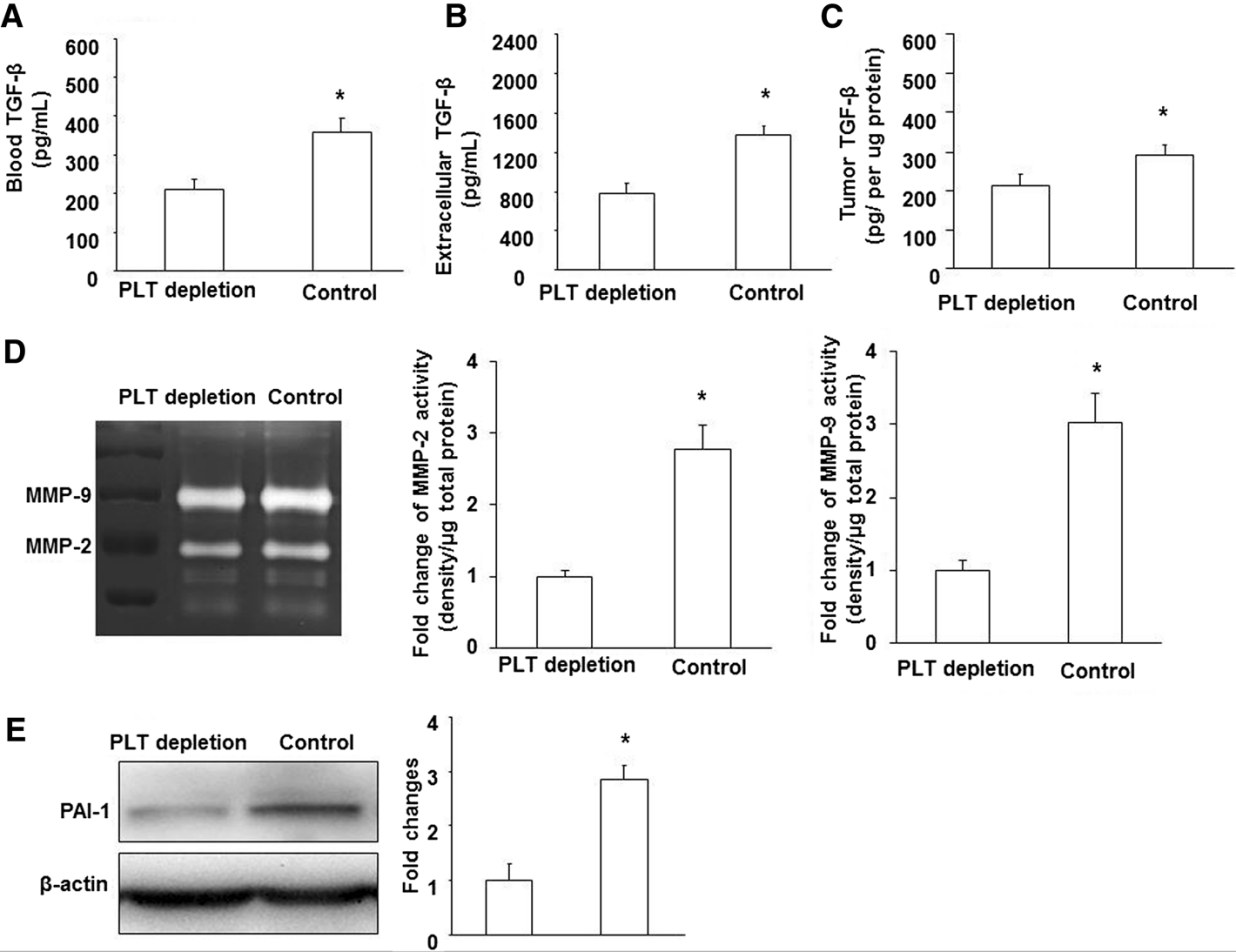
**Platelets changed levels of TGF-β1, MMP-2,9, and PAI-1.** TGF-β1 levels were measured by ELISA in plasma **(A)**, microdialysates **(B)**, and tumor hemogenates **(C)** as described in *Materials and Methods*. **(D)**. Gel zymography analysis of MMP-2, 9 from microdialysates in tumors from PLT-depleted, control mice. **(E)**. Western blot analysis of PAI-1 expression in tumors from PLT-depleted, control mice (normalized to β-actin). The results are expressed as the mean ± SEM. * p < 0.05.

Considering that platelet-derived TGF-β1 plays a key role in regulating circulating cancer cell dissemination [3], we measured the influence of platelets on systemic and local TGF-β1 levels. Blood, extracellular microdialysate, and tumor samples were obtained from platelet-depleted, control, and co-implantation of B16/F10 mice. A significant increase in plasma TGF-β1 from co-implantation of B16/F10 group compared with B16/F10 alone (523 ± 49.75 pg/mL *Vs*. 329 ± 36.27 pg/mL, p < 0.05). The plasma TGF-β1 level of platelet-depleted mice was significantly lower than in control mice (252 ± 25.83 pg/mL *Vs*. 329 ± 36.27 pg/mL, p < 0.05) (Figure 6A). In comparison, an similar findings were found in microdialysates and tumor samples in which the TGF-β1 level of PLT-depletion was significantly lower than in control and co-implantation mice (Figure 6B and C).To better investigate the molecular mechanisms by which platelet depletion reduces metastasis in the tumor microenvironment, we further evaluated the expression of proteins that are involved in regulating the basal membrane and barrier cancer cell intravasion. Zymographic analysis of microdialysates revealed that the intensity of MMP-9 and MMP-2 bands in PLT-depletion were lower than in control groups (Figure 6D). Furthermore, MMP-9 and MMP-2 bands in the co-implantation group were more intense than in the control group. Plasminogen activator inhibitor-1 (PAI-1) protein level was reduced in platelet-depleted tumors compared with control and co-implantation tumors (Figure 6E), suggesting a lower capacity to cross through surrounding microenvironment by degrading several components of the extracellular matrix (ECM).

## Discussion

Many experimental studies using in vitro assays and in vivo metastatic animal models have demonstrated a mechanistic link between tumor cell dissemination and platelet activation. Direct contact between platelets and tumor cells has been observed in the primary tumor microenvironment. Platelet involvement in primary tumor growth and invasiveness has not well been recognized. The process of metastasis initiation includes detachment of tumor cells from the primary site and migration to and intravasation into the blood vessel.

Several angiogenic molecules, including angiopoietins, VEGF, and TGF-β, are abundant in platelets and may affect the tumor microenvironment [1,2,22]. As a Tie2-antagonist, Ang-2 mediates angiogenic sprouting and vascular regression. We found that platelet-depleted tumors exhibited an increase in Ang-2 levels, leading to a delay in vessel maturation and diminished pericyte recruitment to blood vessels that were highly permeable and hemorrhagic. This finding is supported by the recent observation that platelet depletion displayed a significantly lower vessel density and poor vascular maturation in a tumor implantation model and in hindlimb ischemia animal models [6].

Furthermore, it is well known that VEGF plays an important role in the initiation of tumor angiogenesis. Platelet-derived TGF-β is known as an important growth factor involved in circulating dissemination [3]. In fact, TGF-β also provides proliferative signals to tumor cells, which might contribute to the ECM breakdown that is required for vessel invasion to occur [23]. We used microdialysis to examine the extracellular VEGF and TGF-β levels in solid B16/F10 tumors, and the results showed that platelet-depleted mice exhibited a decreased secretion of both VEGF and TGF-β compared to control mice. It should be noted that most serum VEGF is derived from platelets, which are activated upon coagulation [24]. Further studies are required to clarify the role of platelets in the storage of VEGF released from the tumors.

Tumor progression and metastasis are strongly related to blood vessel maturation and stabilization in the tumor microenvironment. Platelets are involved in vessel maturation through multiple mechanisms, including releasing platelet-derived factors and cytokines and regulating bone marrow-derived cell recruitment [5,25,26]. VE-cadherin is crucial for vessel assembly and integrity during angiogenesis [18,27,28]. Likely, increased intratumoral VE-cadherin expression might contribute to vessel lumen development. VE-cadherin also promotes tumor progression not only by contributing to tumor angiogenesis but also by enhancing tumor cell proliferation via the TGF-β1 signaling pathway in breast cancer [29]. Interestingly, we found a significant decrease in VE-cadherin expression in platelet-depleted tumors, suggesting that high VE-cadherin in tumors may lead to an enlarged vessel lumen and is linked to tumor progression in the presence of platelets.

Invasion through the ECM is an important step in tumor metastasis. Cancer cells initiate invasion by adhering to and spreading along blood vessel walls. Proteolytic enzymes, such as MMP, degrade ECM surrounding the blood vessels to allow cancer cells to invade. Alternatively, it is important to note that tumor metastasis is associated with blood vessel maturation and stabilization in the primary tumor. Intravasation of cancer cells does not occur solely though the vessel wall but also through the ECM (basement membrane). TGF-β1 is a crucial factor in inducing tumor growth and metastasis through up-regulating MMP-2, 9. Intratumoral TGF-β1 is constitutively secreted by B16/F10 tumor cells, as well as by direct platelet-tumor cell [30]. We found a significant reduction of TGF-β1 in blood, extracellular space and intracellular tumors from platelet-depleted tumor-bearing mice. Circulating platelet-derived TGF-β1 has been reported to promote metastasis work by activating activate the TGFβ/Smad and NF-kB pathways in cancer cells [3].

Our data demonstrated that platelet depletion reduced metastasis and was further associated with decreased ECM degradation and reduced expression of MMP-2, 9 and PAI-1. The ECM surrounding blood vessels plays a critical role in the limitation of extravasation and intravasation in the tumor microenvironment. Thus, it is possible that platelet-promoted primary tumor metastasis is mainly associated with the integrity of the ECM in the tumor microenvironment as a part of vessel maturation.

Our data demonstrated that Platelet depletion strongly reduced the expression and tyrosine phosphorylation of the Met receptor in tumors. Met expression has been shown to result from increased tumor hypoxia. Our data demonstrated that platelet depletion decreased metastasis and was associated with decreased HIF-1a. It is well documented that tumor hypoxia is associated with vessel structure abnormalities, such as leakiness and destabilization by poor coverage of pericytes, and with excessive proliferation of tumor cells. A recent study demonstrated that platelet depletion causes a decrease in tumor proliferation and delays vessel maturation [7]. Either a change in excessive tumor cell proliferation or impaired vessel maturation accelerates tumor hypoxia. The impaired vessel maturation may lead to an increase in the interstitial pressure due to leakage and thus alter the blood flow because of the compression of tumor vessel, thus likely reduce tumor perfusion. Although we found that platelet depletion significantly reduced blood vessel perfusion of tumors, in this study, the impaired tumor angiogenesis and vessel maturation induced by platelet depletion are not sufficient to cause significant tumor hypoxia. It seems that tumor cell proliferation could play a major role in causing hypoxia in the tumor microenvironment.

Platelet-tumor cell contact promotes the hematogenous dissemination of tumor cells by activating the NF-κB pathway [3]. Abundant platelets were detected in the tumor microenvironment outside of the vasculature [7]. Indeed, a previous study has shown that NF-κB is a key orchestrator of innate immunity/inflammation in many cancers [31]. Labelle et al. identified the involvement of inflammatory cytokines in the platelet-related NF-κB pathway [3]. HIF-1α is an inflammatory response gene. Furthermore, the presence of messengers of inflammation is strong associated with the occurrence of vascular remodeling and angiogenesis. Therefore reduced vessel density and/or function underlie, cannot rule out completely the contribution of immune/inflammatory cells for platelet-induced phenotype.

## Conclusions

In summary, our data provide direct evidence that platelet depletion reduce primary tumor metastasis and are associated with tumor hypoxia, ECM changes and vessel maturation in the tumor microenvironment.

## Abbreviations

H&E: Hematoxylin and eosin; PBS: Phosphate buffered saline; CO2: Carbon dioxide; 3-D: 3-dimention; Col: Collagen; ECM: Extracellular matrix; ECs: Endothelial cells; FITC: Fluorescein isothiocyanate; Ang-1: Angiopoietin-1; Ang-2: Angiopoietin-2; TGF-β: Tumor growth factor-β; VEGF: Vascular endothelial growth factor; WT: Wild type.

## Competing interests

The authors declare that they have no competing interests.

## Authors’ contributions

RL designed research, performed experiments, analyzed data and wrote the manuscript; MR, NC, ML, XD, JX, GY, JL, BH, XZ, ZZ, XZ and BR performed part of the experiments. JW designed the research, supervised the experiments, and edited the manuscript. All authors read and approved the final manuscript.

## Pre-publication history

The pre-publication history for this paper can be accessed here:

http://www.biomedcentral.com/1471-2407/14/167/prepub

## Supplementary Material

Additional file 1 **Platelet depletion showed no change in 4T1 tumor growth and reduced lung metastasis.** (A) Orthotopic implantation of 4T1 mouse mammary epithelial cancer cells into BALB/c mice followed by injections every 3 days of GPI or control antibody after the tumors reached ~500 mm3. (B) Representative images of H&E-stained lung sections. Scale bar, 5 μm. Arrows point to metastatic areas. High-magnification images of metastatic nodules are visualized. Scale bar, 50 μm. (n = 6 for each group). Click here for file
