# Supplementary material for: Presence of intratumoral platelets is associated with tumor vessel structure and metastasis
Source: BMC Cancer. 2014 Mar 10;14:167. doi: 10.1186/1471-2407-14-167 (PMC4016490; doi:10.1186/1471-2407-14-167)
Supplement: Additional file 1 — Platelet depletion showed no change in 4T1 tumor growth and reduced lung metastasis. (A) Orthotopic implantation of 4T1 mouse mammary epithelial cancer cells into BALB/c mice followed by injections every 3 days of GPI or control antibody after the tumors reached ~500 mm3. (B) Representative images of H&E-stained lung sections. Scale bar, 5 μm. Arrows point to metastatic areas. High-magnification images of metastatic nodules are visualized. Scale bar, 50 μm. (n = 6 for each group). [file 1471-2407-14-167-S1.ppt]

## Slide 1
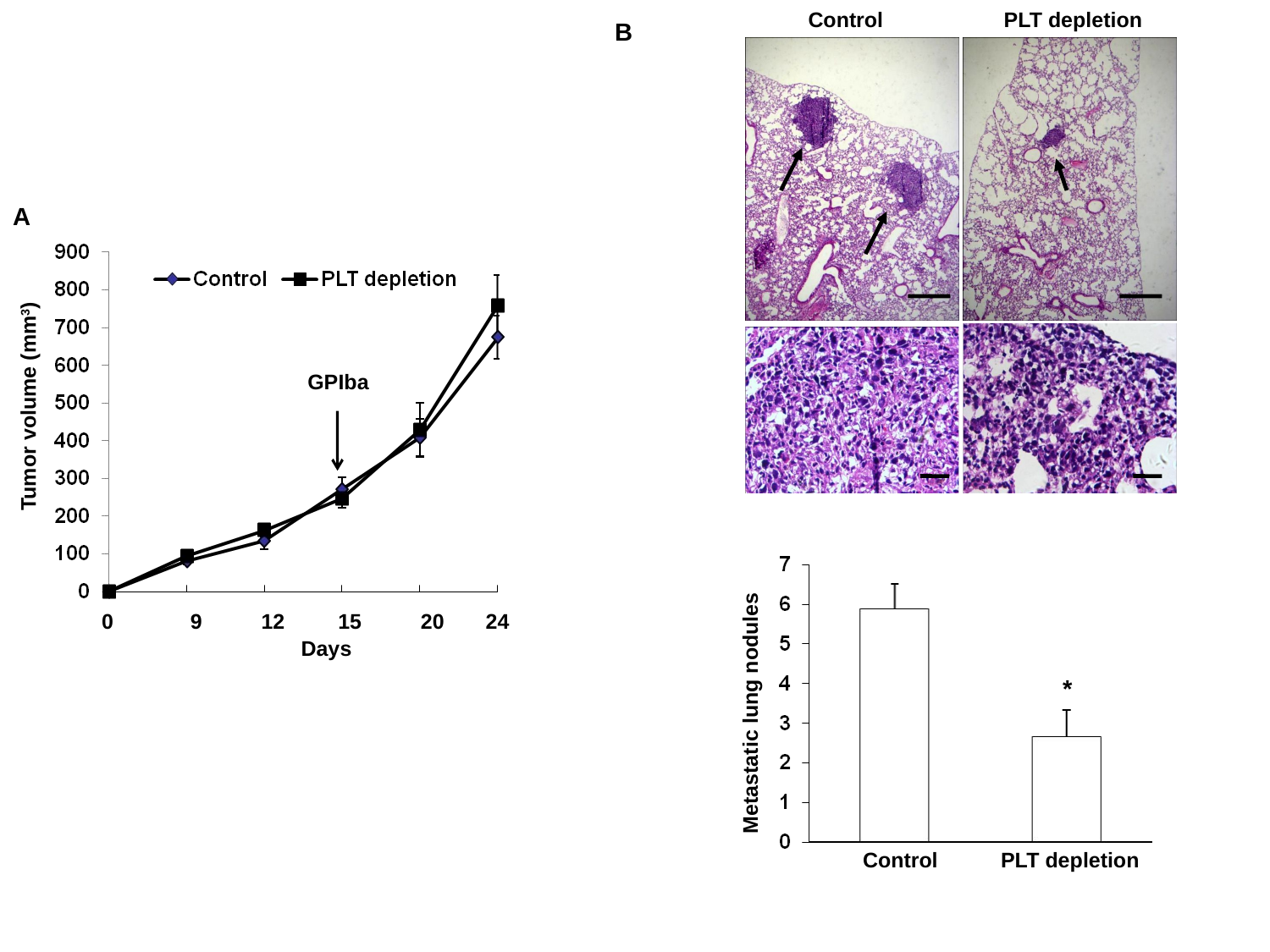

Control
PLT depletion
B
A
GPIba
Tumor volume (mm3)
0 9 12 15 20 24
Days
*
Metastatic lung nodules
Control
PLT depletion
